# Supplementary material for: Knowledge of Human Monkeypox Infection among Final Year Medical, Pharmacy, and Nursing Students: A Multicenter, Cross-Sectional Analysis from Pakistan
Source: Healthcare (Basel). 2023 Oct 20;11(20):2777. doi: 10.3390/healthcare11202777 (PMC10606159; doi:10.3390/healthcare11202777)
Supplement: Supplementary file 1 [file healthcare-11-02777-s001.zip › healthcare-2621169-supplementary.pdf]

Table S1 – Study Questionnaire - knowledge of human monkeypox' source, signs/symptoms, transmission, prevention and treatment among final year medical, pharmacy and nursing students

Section 1 Demographic details

1. Name of medical/pharmacy/nursing college or university…………………………………
2. Age (years) ……………………...
3. Category of student A) Medical B) Pharmacy C) Nursing
4. Types of institute A) Public B) Private
5. Gender: A) Female B) Male
6. Residence A) Rural B) Urban
7. Family income (thousands) A) <25000 B) 25000 to 75000 C) >75000
8. Profession of parents A) Medical (related to health) B) Non-Medical
9. Had you ever received information of human Monkeypox in recent times (last 6 months)?
   A) Yes B) No

If answer is 'No' then submit the study questionnaire,

Section 2 knowledge items about human monkeypox' source, Signs/symptoms, transmission, prevention and treatment

Sr. No.	Questionnaire	Yes	No	Don't Know
1	Monkeypox is a viral infection			
2	Monkeypox is a bacterial infection			
3	Monkeypox occurs primarily in tropical rain-forest areas of Africa and is occasionally exported to others region			
4	Monkeypox and smallpox have similar signs and symptoms			
5	The incubation period of Monkeypox is usually from 3 to 17 days			
6	Monkeypox is easily transmitted from animal-to-human through direct contact with the blood, bodily fluid, cutaneous or mucosal lesions			
7	Monkeypox can be transmitted from animal to human by eating insufficiently cooked meat from an infected animal			
8	Monkeypox can easily be transmitted from human to human through close contact with respiratory secretions, skin lesions of the infected person or contaminated objectives of infected person			
9	Flu-like syndrome is one of the early signs or symptoms of human Monkeypox			
10	Fever along with skin rashes usually begins within 1–3 days are one of the signs or symptoms of human Monkeypox			
11	Papules on the skin are one of the signs or symptoms of human Monkeypox			

12	Vesicles on the skin are one of the signs or symptoms of human Monkeypox			
13	Pustules on the skin are one of the signs or symptoms of human Monkeypox			
14	Lymphadenopathy (swollen lymph nodes) is one signs or symptoms that could be used to differentiate monkeypox and smallpox cases			
15	Fever, Exhaustion, Back and muscle ache and Intense headache are the signs or symptoms of human Monkeypox			
16	Frequent hands washing for at least 20 seconds with soap or alcohol-based hand sanitizers is essential to prevent further human Monkeypox transmission.			
17	Avoiding contact with wild animals (alive or dead) essential to prevent further human Monkeypox transmission.			
18	Monkeypox could be prevented by cooking meat properly			
19	Avoiding contact with any objective that have been in contact with sick animal can prevent spread of Monkeypox			
20	Avoiding contact with any person that has a rash can prevent spread of Monkeypox			
21	Avoiding contact with any objective that has been in contact with sick person can prevent spread of Monkeypox			
22	Reporting symptoms of Monkeypox to local health authorities is important to prevent further disease transmission			
23	Monkeypox usually a self-limited disease with the symptoms lasting from 2 to 4 weeks			
24	Symptomatic supportive care is to be considered in the management of Monkeypox disease			
25	One management option for monkeypox patients who are symptomatic is to use paracetamol			
26	Antibiotics are effective in human Monkeypox			
27	Monkeypox can be treated with the available antiviral medications			

Section 3 Sources of information

From where did you get this information frequently,

1 Print and electronic media

2 social media like Facebook, Twitter, Instagram etc.

3 Smartphone application

4 Journals/books

5 Peers

6 Health care providers

Thank you for your time to provide your response.
